# Perspectives on Europe’s health care systems: meeting future challenges through innovative health care strategies

**DOI:** 10.1186/1878-5085-5-S1-A82

**Published:** 2014-02-11

**Authors:** Karolina Budych, Christoph A Karle, Thomas M Helms

**Affiliations:** 1 German Foundation for the Chronically Ill, Fürth, Germany; 2 Praxis für Diagnostik Hohenlohe, Künzelsau, Germany

## Background: key challenges to Europe’s health care systems

European countries are experiencing significant demographic, epidemiological and health care changes that will shape forthcoming health conditions and challenge the future of health systems [1]. An ageing population and the related rise in chronic disease that require long-term treatment, the costly technological advances and the patient demand driven by increased knowledge of options and higher expectations lead to rising healthcare costs. At the same time, ageing will reduce the economically active population and affect the funding and sustainability of the health and welfare systems in many countries. Spending on healthcare has risen steadily in Europe for the past two decades to an average of about 9% of GDP today [2]. Cardiovascular diseases are a major contributor to total health care costs, the annual cost to the EU is estimated to be over 192 billion Euro [3]. Health inequalities and regional differences in the supply of medical resources create additional demands on the health system and further challenges that go beyond medical problems. Ensuring a multilevel and holistic response by policy makers and by health care providers will be important in tackling these challenges. Innovative health care strategies are needed that provide an answer to growing healthcare costs by delivering greater cost-efficiency and economic productivity.

## Innovative health care strategies: a multifaceted approach to overcome recent and future challenges

Chronic cardiovascular diseases require long-term disease management which can be delivered through self-management and community-based care. A significant role plays the patient himself who can through behavioral change prevent worsening of the disease. Therefore, there is a trend in health care towards a shift in the patients´ role from passive consumers to proactive and well-informed partners in the medical encounter. At the same time, general physicians become more important as gatekeepers to the system and as co-coordinators of treatment for patients with chronic cardiovascular diseases. Hence, new ways of thinking and collaborating in the field of chronic diseases are needed that comprise educational support, shared decision-making and skills development – for both the health care provider and the patient. In this context, e-health can help to facilitate changes in health systems and to promote patient empowerment by providing education and offering opportunities for individualized, tailored healthcare [4]. When combined with organizational change and with the development and implementation of new standards and new qualification offers, e-health can improve healthcare quality and increase equity of access to services and also promote scalability of large public health interventions which could increase cost-efficiency in healthcare delivery. A concerted, multilevel approach is required to develop new processes and structures and to produce sufficient coherence, scale and intensity of actions capable of implementing innovative solutions and supporting the paradigm shift in health care. In order to drive healthcare innovation in an era of budgetary pressure and to achieve technological and political solutions, e-health must be integrated in a network that supports the activity in the regional health care system. The integration of a special telemedical service centre is another prerequisite for a successful implementation of e-health solutions for cardiovascular patients since it enables comprehensive patient care with the centre taking the role of coordinator within the network consisting of general physician, resident cardiologist and hospital. E-health may also help to improve access to medical services that would often not be consistently available in rural communities, where distance is a critical factor. The aim is to offer cardiovascular patients individualized solutions and to implement medical treatment in accordance with the guidelines in order to improve the patients´ quality of life, to prevent hospitalizations and to improve patients’ prognosis. Finally, new information processing technologies allow the integration of collected data into an electronic health record with password protection which can be accessed by different users (general physician, resident cardiologist and physicians at hospital).

## Outlook and recommendations

The rise of chronic diseases presents a major challenge to health and health systems throughout the European Region. Europe’s health care systems need to address this challenge and to manage change, from an acute care towards a chronic disease management, with a focus on prevention and patient-education. This paradigm shift in health and chronic disease management requires educational support for providers and patients in order to increase awareness of chronic diseases. E-health can help patients to become more actively engaged in their own state of health; besides, by changing the role of health care professionals from authority to empowerment and by giving patients access to more information on health care services available, it can optimize the communication between patient and health care provider. This strategy can be combined with other health care solutions focusing on patient empowerment and improving personalized treatment of cardiovascular patients. However, although proven to be technically manageable and of great value in optimizing patient care and increasing efficiency of the health system, the implementation of e-health essentially depends on the acceptance on the part of both patients and physicians. Therefore, future e-health concepts need not only to boost the development of standards and clinical guidelines – given the inescapable trends towards more prevention and patient involvement, future health strategies in Europe should also have a clear patient-centered focus.
